# Hemoglobin glycation index and cardiovascular outcomes in patients with diabetes and coronary artery disease: insights from a large cohort study

**DOI:** 10.1038/s41387-024-00318-x

**Published:** 2024-08-28

**Authors:** Zhangyu Lin, Jining He, Sheng Yuan, Chenxi Song, Xiaohui Bian, Min Yang, Kefei Dou

**Affiliations:** 1 State Key Laboratory of Cardiovascular Disease, Beijing, China; 2 Cardiometabolic Medicine Center, Fuwai Hospital, National Center for Cardiovascular Diseases, Chinese Academy of Medical Sciences and Peking Union Medical College, Beijing, China; 3 Department of Cardiology, Fuwai Hospital, National Center for Cardiovascular Diseases, Chinese Academy of Medical Sciences and Peking Union Medical College, Beijing, China; 4 National Clinical Research Center for Cardiovascular Diseases, Beijing, China

**Keywords:** Cardiovascular diseases, Diabetes complications

## Abstract

**Background/objectives:**

The hemoglobin glycation index (HGI) has been demonstrated to serve as a substitute for the individual bias in glycosylated hemoglobin A1c (HbA1c). Our objective was to assess the correlation between HGI and cardiovascular (CV) outcomes in patients with diabetes and coronary artery disease (CAD).

**Subjects/methods:**

We sequentially recruited 11921 patients with diabetes and CAD at Fuwai Hospital. The patients were categorized into five groups based on their HGI quintiles, ranging from Q1 to Q5. The primary endpoint was the occurrence of major adverse cardiac events (MACEs), which included CV death and nonfatal myocardial infarction.

**Results:**

During the median 3-year follow-up, 327 (2.7%) MACEs were observed. A U-shaped relationship between HGI and 3-year MACEs was demonstrated by restricted cubic spline (RCS) after multivariable adjustment (nonlinear *P* = 0.014). The Kaplan-Meier curves demonstrated that the Q2 group had the lowest risk of MACE (*P* = 0.006). When comparing the HGI Q2 group, multivariable Cox regression models showed that both low (Q1) and high (Q4 or Q5) HGI were linked to a higher risk of MACEs (all *P* < 0.05). Patients with a low HGI (Q1) had a significantly increased risk of all-cause and CV death, with a 1.70-fold increase in both cases (both *P* < 0.05).

**Conclusions:**

In individuals with diabetes and established CAD, HGI levels were found to have a U-shaped relationship with the occurrence of MACEs over a period of three years. Significantly, those with low HGI had an increased risk of CV death.

## Introduction

Globally, cardiovascular (CV) disease, particularly coronary artery disease (CAD), continues to be the primary cause of death [1]. Diabetes is well recognized as a significant contributor and risk factor for CAD [2]. For patients with diabetes and CAD, it is crucial to effectively control blood sugar levels and use reliable indicators, such as glycosylated hemoglobin A1c (HbA1c) to minimize diabetes-related complications and mortality [3]. While HbA1c assays have been standardized, discrepancies between HbA1c and other glycemic assessments have been observed and might affect the accurate interpretation and treatment of glycemic control [4, 5]. Glucose gradient across the red blood cell membrane and red blood cell turnover may affect the HbA1c level independently of glycemia [6]. Therefore, HbA1c levels may deviate from blood sugar levels in a consistent manner owing to several variables that affect the process of glycation inside red blood cells, resulting in HbA1c values that are either lower or higher than anticipated.

To solve these problems, Hempe et al. [7] developed the hemoglobin glycation index (HGI) as a means to assess the variation in individual HbA1c levels. HGI was determined by subtracting the projected HbA1c, based on blood glucose estimates using linear regression, from the observed HbA1c [8]. Presently, there have been few research that examined the correlation between CV outcomes and HGI, and the findings have been rather contentious. Prior research has shown that patients with elevated HGI are more susceptible to developing diabetes-related retinopathy [9], nephropathy [10], and CV complications for patients with diabetes [8]. However, recent studies with limited sample sizes have shown that people with diabetes who have low HGI are more likely to have CV events compared to those with intermediate HGI [11, 12]. Currently, there has been no research conducted to examine the impact of HGI on CV outcomes in individuals who have diabetes and established CAD. Our objective was to examine the correlation between the HGI and the prognosis in individuals diagnosed with both diabetes and CAD.

## Materials/subjects and methods

### Study design and population

This study was a retrospective analysis of a prospective cohort done at Fuwai Hospital, Chinese Academy of Medical Sciences. The study was approved by the Institutional Review Board of Fuwai Hospital and followed the guidelines set forth in the Declaration of Helsinki. All participants provided written informed consent. This research was reported in accordance with the Strengthening the Reporting of Observational Studies in Epidemiology (STROBE) reporting criteria [13].

Between January 2017 and December 2018, a total of 13,506 individuals diagnosed with diabetes and angiography-confirmed CAD had regular post-discharge monitoring. Inclusion criteria were: 1) age ≥ 18 years; 2) participants had to have angiography results confirming the presence of CAD, with at least one coronary artery showing a stenosis of 50% or more; 3) participants had to have a confirmed diagnosis of diabetes. Diabetes was diagnosed if the patient had a previous diagnosis of diabetes, was on treatment to decrease glucose levels, or had a fasting blood glucose (FBG) level of 7.0 mmol/L or higher, HbA1c level of 6.5% or higher, or a 2-hour plasma glucose level of 11.1 mmol/L or higher during an oral glucose tolerance test [3]. Exclusion criteria were: 1) absence of important laboratory data; 2) severe liver or renal malfunction; 3) decompensated heart failure; 4) systemic inflammatory illness; 5) malignant tumor; 6) anemia; 7) hematological disorder; and 8) patient who lost to follow-up. Finally, 11921 individuals diagnosed with both diabetes and CAD were included in this study (Fig. S1).

### Deriving HGI from the HbA1c Versus FBG regression equation

The calculation of HGI was performed using the methods proposed by Hempe et al. [8]. Briefly, the baseline FBG and HbA1c data of all individuals were used to assess the linear relationship between FBG and HbA1c in the study group. (Fig. S2). Subsequently, a predicted HbA1c value was calculated by combining the baseline FBG with the subsample linear regression equation (predicted HbA1c = 0.013 × FBG [mmol/L] + 5.455). The baseline HGI was calculated by subtracting the expected HbA1c from the actual HbA1c. All participants would be classified based on their baseline HGI quintiles (quintile 1 [Q1]: ≤ −0.840; Q2: −0.840 to −0.322; Q3: −0.322 to 0.075; Q4: 0.075 to 0.790; Q5: ≥ 0.790).

### Data collection and definitions

Demographic and clinical information for all patients was obtained prospectively. The demographic data collected consisted of age, sex, body mass index (BMI), presence of concomitant disorders, smoking status, family history of CAD, history of prior myocardial infarction (MI), and history of percutaneous coronary intervention (PCI) or coronary artery bypass grafting (CABG). The clinical data included the primary diagnosis at admission, the results of physical, radiological, and laboratory examinations, and the prescribed drug regimen upon discharge.

Upon admission, we obtained laboratory samples from each participant by drawing blood from the cubital vein after a minimum of 12 h of fasting. Our center’s clinical chemistry branch conducted all the exams. An enzymatic test was used to assess the amounts of triglycerides (TG), total cholesterol (TC), high-density lipoprotein cholesterol (HDL-C), FBG, and creatinine. The analysis was performed using an automated biochemical analyzer (Hitachi 7150, Tokyo, Japan). The calculation of low-density lipoprotein cholesterol (LDL-C) was performed using the Friedewald technique [14]. HbA1c was determined using high-performance liquid chromatography (Tosoh G8 HPLC Analyzer; Tosoh Bioscience, Tokyo, Japan). The high sensitivity C reactive protein (hsCRP) was analyzed using conventional biochemical methods at the central laboratory of Fuwai Hospital. The estimated glomerular filtration rate (eGFR) was determined using the Chinese-modified Modification of Diet in Renal Disease equation [15]. The modified biplane Simpson rule was used to evaluate the left ventricular ejection fraction (LVEF) at a state of rest [16].

The procedure of coronary angiography was carried out using standard techniques by experienced interventional cardiologists. Two experienced interventional cardiologists, working separately, examined the angiographic data obtained from the catheter laboratory at Fuwai Hospital. They documented the specific features of CAD, including unique types of narrowing in the coronary arteries, as well as the SYNergy between percutaneous coronary intervention with TAXus and cardiac surgery (SYNTAX) score.

Hypertension was characterized as having a systolic blood pressure (SBP) equal to or more than 140 mmHg, a diastolic blood pressure (DBP) equal to or greater than 90 mmHg, or the use of antihypertensive treatment [17]. Chronic kidney disease (CKD) was defined as the eGFR < 60 mL/min/1.73 m^2^ persisting for a duration of at least 3 months [18].

### Follow‐up and study endpoints

Participants were monitored at 6-month intervals until December 31, 2021, after their discharge. The data for endpoints were collected from medical records, clinical visits, and/or telephone interviews by experienced investigators who were unaware of the study design. The primary endpoint was the major adverse cardiac event (MACE), which included CV death and nonfatal myocardial infarction (MI). The secondary endpoints included all-cause death, CV death, nonfatal MI, and unplanned revascularization. Death was classified as CV-related unless a clear non-CV cause could be determined. Nonfatal MI was defined as the presence of positive cardiac troponins together with typical chest pain, characteristic electrocardiogram serial alterations, identification of an intracoronary thrombus by angiography or autopsy, or imaging data indicating fresh loss of viable myocardium or a new regional wall-motion abnormality [19]. Unplanned revascularization refers to the need for a treatment to restore blood flow to a lesion that did not satisfy the threshold for ischemia during the first operation and was not intended to be treated with a subsequent planned revascularization. Two separate physicians meticulously assessed all events.

### Statistical analysis

Continuous variables were presented as mean ± standard deviation (SD) or median (interquartile range, IQR), and were compared using either the Student t-test or the Mann-Whitney U test. Categorical variables were represented as numerical values and percentages, and were compared using either the Fisher’s exact test or the chi-square test. The Kaplan-Meier curves were used to demonstrate the cumulative incidence of clinical endpoints across different groups, and the log-rank test was then used to compare these incidences. After adjusting for age and sex, we used restricted cubic spline (RCS) models to examine the presence of nonlinearity between continuous HGI and the likelihood of experiencing MACE during a 3-year period. The hazard ratios (HRs) and 95% confidence intervals (CIs) were calculated using both univariable and multivariable Cox regression models. The multivariable Cox regression model used age, sex, BMI, duration of diabetes, acute coronary syndrome (ACS) presentation, family history of CAD, MI histories, previous revascularization, hypertension, previous stroke, PAD, current smoker, LVEF, serum creatinine, TG, LDL-C, HDL-C, TC, hsCRP, LM/three-vessel disease, chronic total occlusion (CTO) lesion, ostial lesion, type B2/C lesion, severe calcification, aspirin use, statins use, and insulin use as covariates. A two-tailed P value less than 0.05 was considered to be statistically significant. The analyses were performed using R version 4.0.3 software (R Foundation, Vienna, Austria).

## Results

### Baseline characteristics according to HGI quintiles

A total of 11921 patients were ultimately included. The mean age was 60.69 ± 9.78 years, and 8955 (75.1%) were male. Additionally, 9014 (75.6%) patients had hypertension, and 3639 (30.5%) were currently smoking (Table 1). The participants were divided into five groups based on their HGI quintiles: Q1 (*N* = 2384), Q2 (*N* = 2375), Q3 (*N* = 2389), Q4 (*N* = 2387), and Q5 (*N* = 2386). The detailed baseline data for each group can be found in Table 2. In general, individuals with a low HGI (Q1) had a higher likelihood of being diagnosed with ACS and having a history of MI, CKD, poorer LVEF, higher serum creatinine levels, and higher SYNTAX scores. These patients also had more thrombotic lesions, ostial lesions, and type B2/C lesions. Patients with high HGI (Q5) exhibited elevated levels of HbA1c, FBG, TC, LDL-C, and were prescribed more glucose-lowering medications.

**Table 1:** Baseline characteristics according to HGI quintiles.

Characteristics^a^	Overall N = 11921	HGI quintiles					*P* value
		Q1, <−0.840 *N* = 2384	Q2, [−0.840, −0.322) *N* = 2375	Q3, [−0.322, 0.075) *N* = 2389	Q4, [0.075, 0.79) N = 2387	Q5, ≥ 0.79 *N* = 2386	
HGI, %	−0.14 (−0.68, 0.54)	−1.29 (−1.67, −1.03)	−0.54 (−0.68, −0.42)	−0.14 (−0.23, −0.04)	0.36 (0.20, 0.54)	1.44 (1.08, 2.07)	<0.001
Age, years	60.69 ± 9.78	60.51 ± 10.12	60.69 ± 9.53	60.95 ± 9.66	61.00 ± 9.68	60.30 ± 9.91	0.075
Male	8955 (75.1)	1905 (79.9)	1831 (77.1)	1767 (74.0)	1748 (73.2)	1704 (71.4)	<0.001
BMI, kg/m^2^	26.30 ± 3.23	25.91 ± 3.18	26.29 ± 3.13	26.37 ± 3.22	26.42 ± 3.21	26.52 ± 3.40	<0.001
Duration of Diabetes	6 (0, 10)	1 (0, 9)	5 (1, 10)	3 (0, 9)	7 (1, 10)	9 (5, 15)	<0.001
Clinical presentation							<0.001
CCS	4222 (35.4)	670 (28.1)	879 (37.0)	894 (37.4)	920 (38.5)	859 (36.0)	
ACS	7699 (64.6)	1714 (71.9)	1496 (63.0)	1495 (62.6)	1467 (61.5)	1527 (64.0)	
Family history of CAD	1401 (11.8)	307 (12.9)	299 (12.6)	256 (10.7)	276 (11.6)	263 (11.0)	0.081
Previous MI	3374 (28.3)	782 (32.8)	615 (25.9)	607 (25.4)	692 (29.0)	678 (28.4)	<0.001
Previous revascularization^b^	3734 (31.3)	756 (31.7)	747 (31.5)	732 (30.6)	779 (32.6)	720 (30.2)	0.396
Hypertension	9014 (75.6)	1785 (74.9)	1803 (75.9)	1816 (76.0)	1843 (77.2)	1767 (74.1)	0.113
Previous stroke	1746 (14.6)	358 (15.0)	374 (15.7)	317 (13.3)	354 (14.8)	343 (14.4)	0.172
PAD	863 (7.2)	151 (6.3)	191 (8.0)	159 (6.7)	192 (8.0)	170 (7.1)	0.068
Current smoker	3639 (30.5)	709 (29.7)	697 (29.3)	705 (29.5)	772 (32.3)	756 (31.7)	0.071
CKD	327 (2.7)	88 (3.7)	62 (2.6)	61 (2.6)	60 (2.5)	56 (2.3)	0.034
LVEF, %	61.06 ± 7.20	59.93 ± 7.78	61.65 ± 7.03	61.61 ± 6.75	61.31 ± 7.06	60.78 ± 7.19	<0.001
SBP, mmHg	131.51 ± 17.89	129.74 ± 18.08	131.26 ± 17.20	131.64 ± 17.88	132.47 ± 17.70	132.45 ± 18.37	<0.001
DBP, mmHg	77.19 ± 10.85	77.34 ± 11.31	77.27 ± 10.65	77.09 ± 11.03	77.19 ± 10.57	77.07 ± 10.71	0.903
**Laboratory tests**							
Serum creatinine, μmol/L	82.79 ± 20.32	83.79 ± 22.05	82.69 ± 18.01	82.80 ± 24.01	82.91 ± 18.26	81.78 ± 18.51	0.019
eGFR, mL/min/1.73 m^2^	85.77 ± 19.38	86.64 ± 20.83	86.05 ± 19.15	85.23 ± 17.88	84.76 ± 19.05	86.18 ± 19.82	0.005
HbA1c, %	7.38 ± 1.32	6.16 ± 0.74	6.72 ± 0.61	7.04 ± 0.56	7.74 ± 0.71	9.24 ± 1.14	<0.001
HbA1c, mmol/mol	57.15 ± 14.39	43.86 ± 8.05	49.91 ± 6.67	53.41 ± 6.09	61.07 ± 7.78	77.48 ± 12.49	<0.001
FBG, mmol/L	8.25 ± 2.95	9.04 ± 3.36	7.76 ± 2.57	7.33 ± 2.31	8.13 ± 2.82	8.96 ± 3.17	<0.001
FBG, mg/dL	148.41 ± 53.07	162.72 ± 60.47	139.69 ± 46.27	131.90 ± 41.66	146.42 ± 50.68	161.33 ± 57.08	<0.001
TG, mmol/L	1.51 (1.13, 2.13)	1.49 (1.10, 2.13)	1.47 (1.11, 2.10)	1.54 (1.15, 2.18)	1.54 (1.15, 2.15)	1.50 (1.13, 2.13)	0.037
TC, mmol/L	3.98 ± 0.99	3.88 ± 0.88	3.96 ± 1.02	4.01 ± 0.97	4.00 ± 1.00	4.03 ± 1.06	<0.001
HDL-C, mmol/L	1.07 ± 0.26	1.07 ± 0.24	1.08 ± 0.27	1.08 ± 0.27	1.06 ± 0.26	1.06 ± 0.26	0.009
LDL-C, mmol/L	2.37 ± 0.83	2.29 ± 0.75	2.35 ± 0.86	2.38 ± 0.83	2.39 ± 0.84	2.42 ± 0.87	<0.001
hsCRP, mg/L	1.59 (0.75, 3.37)	1.37 (0.63, 3.15)	1.43 (0.65, 2.95)	1.55 (0.74, 3.30)	1.78 (0.92, 3.71)	1.77 (0.85, 3.76)	<0.001
**Angiographic findings**							
SYNTAX score	12 (7, 19)	12.5 (7, 20)	11 (7, 18)	12 (7, 19)	13 (7, 20)	12 (7, 20)	0.018
LM/three-vessel disease	5989 (50.2)	1109 (46.5)	1185 (49.9)	1209 (50.6)	1242 (52.0)	1244 (52.1)	0.001
CTO lesion	1243 (10.4)	212 (8.9)	263 (11.1)	231 (9.7)	273 (11.4)	264 (11.1)	0.016
Thrombotic lesion	467 (3.9)	250 (10.5)	69 (2.9)	35 (1.5)	49 (2.1)	64 (2.7)	<0.001
Ostial lesion	1451 (12.2)	311 (13.0)	269 (11.3)	252 (10.5)	330 (13.8)	289 (12.1)	0.004
Type B2/C lesion	8878 (74.5)	1819 (76.3)	1725 (72.6)	1780 (74.5)	1783 (74.7)	1771 (74.2)	0.073
Severe calcification	422 (3.5)	77 (3.2)	82 (3.5)	85 (3.6)	92 (3.9)	86 (3.6)	0.836
**Medications**							
Aspirin	8438 (70.8)	1543 (64.7)	1736 (73.1)	1727 (72.3)	1725 (72.3)	1707 (71.5)	<0.001
Statins	11576 (97.1)	2337 (98.0)	2309 (97.2)	2308 (96.6)	2318 (97.1)	2304 (96.6)	0.018
ACE inhibitor or ARB	3262 (27.4)	594 (24.9)	656 (27.6)	659 (27.6)	718 (30.1)	635 (26.6)	0.002
β-Blocker	10713 (89.9)	2143 (89.9)	2107 (88.7)	2127 (89.0)	2159 (90.4)	2177 (91.2)	0.025
CCB	4308 (36.1)	787 (33.0)	898 (37.8)	861 (36.0)	896 (37.5)	866 (36.3)	0.005
Glucose-lowering therapy							
Diet control	1023 (8.6)	224 (9.4)	303 (12.8)	200 (8.4)	179 (7.5)	117 (4.9)	<0.001
Oral medication	5886 (49.4)	870 (36.5)	1256 (52.9)	1086 (45.5)	1306 (54.7)	1368 (57.3)	<0.001
Insulin use	1991 (16.7)	204 (8.6)	303 (12.8)	284 (11.9)	460 (19.3)	740 (31.0)	<0.001

^a^Values are presented as mean ± standard deviation, median (interquartile range), or n (%).

^b^Revascularization included percutaneous coronary intervention and coronary artery bypass grafting.

*ACE* angiotensin-converting enzyme, *ACS* acute coronary syndrome, *ARB* angiotensin receptor blocker, *BMI* body mass index, *CAD* coronary artery disease, *CCB* calcium channel blocker, *CCS* chronic coronary syndrome, *CKD* chronic kidney disease, *CTO*, chronic total occlusion, *DBP* diastolic blood pressure, *eGFR* estimated glomerular filtration rate, *FBG* fasting blood glucose, *HbA1c* glycosylated hemoglobin A1c, *HDL-C* high-density lipoprotein cholesterol, *HGI* hemoglobin glycation index, *hsCRP* high-sensitivity C-reactive protein, *LDL-C* low-density lipoprotein cholesterol, *LM* left main, *LVEF* left ventricular ejection fraction, *MI* myocardial infarction, *PAD* peripheral artery disease, *SBP* systolic blood pressure, *SYNTAX* synergy between PCI with taxus and cardiac surgery; *TC* total cholesterol, *TG* triglyceride.

**Table 2:** Multivariable Cox regression models for HGI quintiles in relation to adverse CV events at 3-year follow-up.

Endpoints^a,b^	Q1, <−0.840	Q2, [−0.840, −0.322)	Q3, [−0.322, 0.075)	Q4, [0.075, 0.790)	Q5, ≥ 0.790
**Model 1**^**c**^					
MACE ^d^	1.73 (1.20–2.49)**	Reference	1.13 (0.76–1.67)	1.64 (1.13–2.36)**	1.64 (1.13–2.36)**
CV death	2.04 (1.34–3.12)***	Reference	1.03 (0.63–1.67)	1.53 (0.98–2.39)	1.60 (1.03–2.48)*
All-cause death	1.96 (1.37–2.80)***	Reference	1.11 (0.75–1.66)	1.52 (1.05–2.21)*	1.48 (1.02–2.15)*
Nonfatal MI	1.01 (0.48–2.11)	Reference	1.35 (0.68–2.69)	1.87 (0.97–3.57)	1.64 (0.85–3.21)
Unplanned revascularization	1.03 (0.82–1.31)	Reference	1.14 (0.90–1.43)	1.35 (1.08–1.68)**	1.39 (1.12–1.74)**
**Model 2** ^**e**^					
MACE	1.50 (1.04–2.17)*	Reference	1.14 (0.77–1.71)	1.57 (1.08–2.27)*	1.50 (1.03–2.18)*
CV death	1.70 (1.10–2.62)*	Reference	1.06 (0.64–1.74)	1.51 (0.96–2.37)	1.56 (0.99–2.45)
All-cause death	1.70 (1.19–2.45)**	Reference	1.19 (0.79–1.78)	1.56 (1.07–2.28)*	1.48 (1.01–2.17)*
Nonfatal MI	1.00 (0.47–2.11)	Reference	1.37 (0.69–2.74)	1.70 (0.88–3.26)	1.37 (0.70–2.70)
Unplanned revascularization	1.11 (0.88–1.41)	Reference	1.16 (0.92–1.46)	1.29 (1.03–1.61)*	1.26 (1.01–1.58)*

^a^Values are presented as hazard ratio (95% confidential intervals).

^b^ns, *P* > 0.05; *, *P* ≤ 0.05; **, *P* ≤ 0.01; *** *P* ≤ 0.001; **** *P* ≤ 0.0001; Bold value indicated statistical significance.

^c^Model 1 was unadjusted.

^d^MACE was defined as a composite of CV death and nonfatal MI.

^e^Model 2 adjusted for age, sex, BMI, duration of diabetes, ACS presentation, family history of CAD, MI histories, previous revascularization, hypertension, previous stroke, PAD, current smoker, LVEF, serum creatinine, TG, LDL-C, HDL-C, TC, hsCRP, LM/three-vessel disease, CTO lesion, ostial lesion, type B2/C lesion, severe calcification, aspirin use, statins use, and insulin use.

MACE, major adverse cardiovascular event; CV, cardiovascular; MI, myocardial infarction.

The Spearman correlation analysis revealed a high positive association between the HGI and HbA1c values (β = 0.845, *P* < 0.001). In addition, the HGI exhibited a positive correlation with conventional CV risk variables, including BMI, duration of diabetes, LVEF, SBP, TC, LDL-C, and hsCRP, and inversely correlated with serum creatinine, eGFR, and HDL-C (Table S1). The distribution of HbA1c varied significantly across the quantiles of HGI, although the distribution of FBG was consistent throughout the HGI quantiles (Fig. S3).

### HGI and adverse CV events risk

**Fig. 1:**
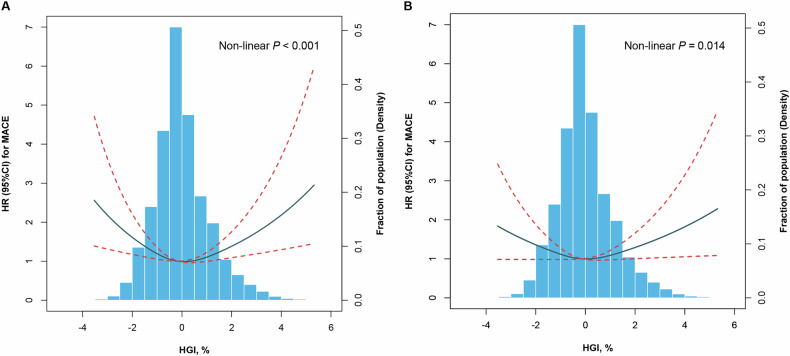
Association between HGI and 3-year MACE. **A** HGI and 3-year MACE in the univariable model; **B** HGI and 3-year MACE in the multivariable model adjusted for adjusted for age, sex, BMI, duration of diabetes, ACS presentation, family history of CAD, MI histories, previous revascularization, hypertension, previous stroke, PAD, current smoker, LVEF, serum creatinine, TG, LDL-C, HDL-C, TC, hsCRP, LM/three-vessel disease, CTO lesion, ostial lesion, type B2/C lesion, severe calcification, aspirin use, statins use, and insulin use. MACE was defined as a composite of CV death and nonfatal MI. ACS, acute coronary syndrome; BMI, body mass index; CAD, coronary artery disease; CI, confidence interval; CTO, chronic total occlusion; CV, cardiovascular HDL-C, high-density lipoprotein cholesterol; HGI, hemoglobin glycation index; HR, hazard ratio; hsCRP, high-sensitivity C-reactive protein; LDL-C, low-density lipoprotein cholesterol; LM, left main; LVEF, left ventricular ejection fraction; MACE, major adverse cardiac events; MI, myocardial infarction; PAD, peripheral artery disease; TC total cholesterol; TG, triglyceride.

**Fig. 2:**
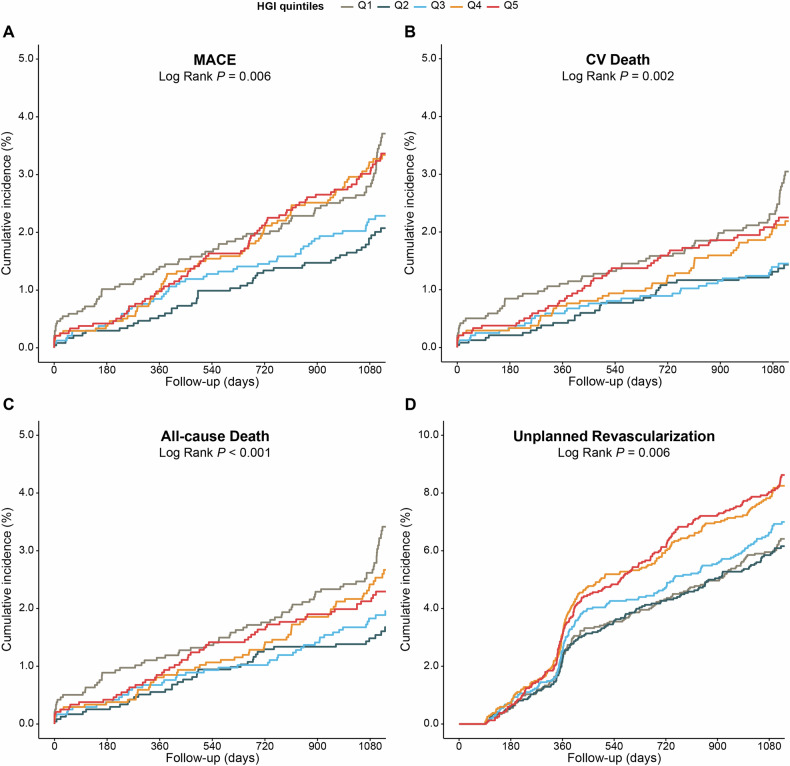
Kaplan-Meier curves of HGI quintiles for clinical outcomes. (**A**) CV Death, (**B**) All-cause Death, (**C**) CV Death and Nonfatal MI, and (**D**) CV Death, Nonfatal MI and Unplanned Revascularization. Abbreviations as in Fig. 1.

Over the course of the 3-year follow-up period, a total of 327 MACEs were documented (Table S2). The incidence of MACEs in the HGI Q1 to Q5 groups were 79 (3.3%), 46 (1.9%), 52 (2.2%), 75 (3.1%), and 75 (3.1%) correspondingly. RCS showed a U-shaped relationship between HGI and 3-year MACEs after multivariable adjustment (non-linear *P* = 0.014, Fig. 1). Kaplan-Meier survival analyses revealed a statistically significant disparity in the occurrence of MACEs among the five groups after a 3-year follow-up. Notably, the HGI Q2 group exhibited the lowest rates of MACEs (all *P* values < 0.001, Fig. 2).

The findings of univariable and multivariable Cox regression models were displayed in Table 2. Results of the multivariable Cox regression analyses indicated that, when compared to subjects in the HGI Q2 group, subjects in the HGI Q1 group had a higher risk of MACEs, CV death, and all-cause death at the 3-year follow-up. The adjusted HR were 1.50 (95% CI: 1.04–2.17), 1.70 (95% CI: 1.10–2.62), and 1.70 (95% CI: 1.19–2.45) for MACEs, CV death, and all-cause death, respectively. Patients categorized in the high HGI quartiles (Q4 and Q5) had a significantly greater risk of MACEs, all-cause death, and unplanned revascularization compared to patients in the Q2 of HGI (all *P* < 0.05). There was no noticeable disparity in the risk of any MACEs between patients in the second and third quartiles of HGI.

## Discussion

This study examined the relationship between HGI and CV outcomes over a period of three years in patients with diabetes and existing CAD. The major discoveries are as follows: 1) Patients with diabetes and CAD exhibit significant variations in their clinical and angiographic characteristics based on their HGI quintiles. Those with low HGI levels have a higher prevalence of comorbid risk factors and more complex coronary lesions, leading to more acute clinical presentations. 2) RCS analysis indicates a U-shaped relationship between HGI and 3-year MACEs. 3) Multivariable Cox regression models demonstrate that both low and high HGI levels are associated with an increased risk of MACE or all-cause death during the 3-year follow-up period. 4) The presence of low HGI primarily contributes to elevated CV and all-cause mortality rates, while higher HGI levels are associated with an increase in unplanned revascularization rates. 5) There is a strong correlation between HGI and HbA1c levels (β = 0.845), suggesting that including HbA1c in an analysis alongside HGI may introduce statistical spuriousness. Our findings initially validated the potential of HGI in mitigating errors in assessing and treating blood glucose levels, which can occur when relying solely on HbA1c and leading to underestimation or overestimation. A high HGI indicates that the HbA1c level reflects a greater blood glucose level than what is actually present. As a result, patients with a high HGI may receive excessive hypoglycemic medication and face an increased risk of hypoglycemia. People with a low HGI have a blood glucose level, as measured by HbA1c, that is lower than their actual blood glucose level. As a result, these persons may not receive the appropriate glucose-lowering treatment and are at a higher risk of experiencing problems related to diabetes. The measurement of HGI is helpful for doctors and their patients as it allows for personalized clinical care and helps prevent harm that may result from incorrect evaluation of glycemic levels and therapeutic management.

Debates continue to exist regarding the influence of glycemic control on CV events for individuals with diabetes. Although several CV outcome trials, such as VADT [20], ACCORD [21], and ADVANCE [22], have not demonstrated a significant decrease in the risk of CV events with more rigorous glycemic control compared to standard care for diabetes, other studies like DCCT/EDIC [23] and UKPDS [24] have suggested a potential advantage of strict glycemic management in reducing the occurrence of CV events. Identifying patients who would benefit the most from glycemic management could offer useful understanding of this matter, and various innovative biomarkers have been created to assist in guiding glycemic management for individuals with diabetes [25]. An example of such a biomarker is the triglyceride-glucose (TyG) index, which has been acknowledged for its ability to predict the occurrence and prognosis of individuals with diabetes and CAD [25]. A recent study has discovered a more pronounced connection between glycemic control status and adverse CV events in persons with elevated TyG levels. This suggests that the TyG index could be helpful in assessing the risk of patients with diabetes and CAD [25]. Our study revealed that HGI can serve as a valuable biomarker in preventing errors resulting from relying solely on HbA1c to assess blood glucose levels in individuals with diabetes and CAD from a different standpoint. HGI might help to avoid underestimation or overestimation of blood glucose levels during glycemic treatment.

The prevailing methods for glycemia management in individuals with diabetes primarily rely on HbA1c [26, 27]. Although assays of HbA1c have been standardized, there are still differences between HbA1c and other glycemic measurements that have been extensively established. These differences have the potential to affect how glycemic control is interpreted and treated [4, 5, 28]. The HbA1c measurement is affected by various factors related to the lifespan and turnover of red blood cells, as well as the glucose concentration across the red blood cell membrane, regardless of blood sugar levels [29, 30]. In order to address these issues, the HGI was created to quantify the discrepancy between HbA1c levels and blood glucose measurements [31, 32]. It is worth mentioning that HGI remains constant in individuals over time. Hempe et al. [7] collected blood glucose data from 128 patients with type 1 diabetes over a period of two years. During the study period, it was observed that HbA1c and blood glucose levels consistently exhibited the same pattern and degree of high glycemic index (HGI), suggesting a consistent fluctuation in intracellular glycation compared to extracellular glycation or blood glucose levels.

Prior research has predominantly shown that elevated levels of HGI are linked to unfavorable prognosis and increased CV risk in individuals with diabetes [32–34]. According to Van Steen et al. [33], there is a positive correlation between high HGI and increased occurrence of microvascular and macrovascular problems, as well as death, in individuals with diabetes. Rajendran et al. [34] found that the association between lipid profile (TC, TG, and LDL-C) and HGI is not influenced by FBG levels in the extra vascular compartment. Our research revealed that there is a direct relationship between HGI and lipid profiles related to CV disease in patients with diabetes and established CAD. This indicates that patients with high HGI are at a greater risk of developing CV complications.

However, recent studies that have employed a more detailed categorization of HGI levels have indicated that the linear association may not truly represent the connection between HGI and CV outcomes [11, 12]. Wang et al. [12] discovered a U-shaped relationship between the levels of HGI and the risk of MACE in patients with diabetes. They observed that both the lowest (Q1) and highest (Q5) HGI levels are associated with a greater risk of MACE compared to the middle (Q2) group. Pan et al. [11] discovered a U-shaped correlation between the HGI and the likelihood of experiencing a stroke within 12 months. This indicates that both low and high HGIs were linked to adverse CV outcomes. Consistent with other research, we observed that HGI levels exhibited a U-shaped correlation with the incidence of MACE during a three-year period in individuals who have both diabetes and established CAD. The risk of death, particularly CV death, considerably rose when levels of HGI decreased.

A low HGI score suggests that there is less glycation than what was initially expected. HGI is intended to measure the difference between the actual and expected values of glycated HbA1c. The results of the ACCORD study indicated that intensive therapy was associated with a decreased likelihood of adverse CV events in the low HGI subgroups (HR = 0.75; 95% CI: 0.59–0.95), but not in the high HGI subgroup (HR = 1.14; 95% CI: 0.93–1.40) [21]. In addition, our research revealed that individuals with low HGI had a greater prevalence of CKD and elevated blood creatinine levels. This suggests a potential correlation between low HGI and an increased occurrence of diabetic macrovascular complications. Thus, additional future research is necessary to examine whether patients with low HGI values can derive advantages from intensive glycemic management.

Notably, our study revealed a higher occurrence of complex coronary lesions, such as thrombotic lesions and ostial lesions, among the groups with lower HGI values and higher SYNTAX scores. Conversely, individuals with low HGI levels exhibited a higher frequency of ACS presentations accompanied by a deterioration in cardiac function. Prior data has indicated that those with more unfavorable CV risk profiles and a higher degree of complexity in CAD are at an elevated risk of experiencing CV events [35]. Further investigations are needed to explore the particular mechanisms linking HGI levels and lesion severity.

This study, which has the largest sample size up to now, initially validated the U-shaped correlation between HGI values and adverse CV outcomes in individuals with diabetes and existing CAD. Furthermore, we have included patients with angiography-proven CAD for the first time. Our study reveals that the angiographic profile differs significantly among different HGI quintiles. Specifically, individuals with low HGI values exhibit a higher complexity of coronary lesions and more acute clinical presentations. Furthermore, we observed that individuals with diabetes and CAD who had low HGI values experienced a greater likelihood of all-cause and CV death.

Nevertheless, this study has several limitations. Firstly, because of the inherent characteristics of the single center and observational study design, it is possible that potential confounding factors may not be completely eliminated. Secondly, we employed FBG as a means of determining HGI, rather than relying on average plasma glucose measurements. Nevertheless, FBG is more readily obtainable in practical scenarios when compared to average plasma glucose. Previous research has demonstrated a noteworthy association between FBG and average plasma glucose levels [34]. Meanwhile, research has demonstrated that the use of FBG to compute HGI is more effective in accurately reflecting the changes in HbA1c levels [34]. Finally, given the linear association between the HGI and HbA1c level, it is impossible to exclude the potential correlation between the effects of the HGI and the HbA1c level. While the HGI is a reliable measure, it is subject to periodic fluctuations. Determining the ideal timing for HGI testing is important.

In conclusion, this large cohort study first confirmed a U-shaped relationship between HGI and the occurrence of MACE within a three-year period in patients with diabetes and established CAD. Both low and high HGI were found to be linked to a greater risk of MACE at the 3-year follow-up. Additionally, patients with low HGI had a higher chance of CV death. These findings imply that HGI could be useful in assessing the risk and prognosis of this particular population.

### Supplementary information

Supplementary Materials

## Data Availability

Data underlying this article and the associated analysis code will be shared upon reasonable request and in accordance with the appropriate general data protection regulation (GDPR).
