# Supplementary material for: Hemoglobin glycation index and cardiovascular outcomes in patients with diabetes and coronary artery disease: insights from a large cohort study
Source: Nutr Diabetes. 2024 Aug 28;14:69. doi: 10.1038/s41387-024-00318-x (PMC11349977; doi:10.1038/s41387-024-00318-x)

**Hemoglobin glycation index and cardiovascular outcomes in patients with diabetes mellitus and coronary artery disease: insights from a large cohort study**

**Supplementary Materials**

**Table of Contents**

[Table S1 Correlation between HGI and clinical risk factors 2](#_Toc146547856)

[Table S2 Rates of 3-Year cardiovascular outcomes stratified by HGI quintiles 3](#_Toc146547857)

[Figure S1 study flowchart 4](#_Toc146547859)

[Figure S2 scatter plot between 5](#_Toc146547860)

[Figure S3 distribution 6](#_Toc146547861)

# Table S1-Correlation between HGI and clinical CV risk factors

| **Variables** | **Correlation coefficient (r)** | ***P* value** |
| --- | --- | --- |
| Age, years | 0.001 | 0.833 |
| BMI, kg/m^2^ | 0.055 | <0.001 |
| Duration of diabetes, years | 0.283 | <0.001 |
| LVEF, % | 0.023 | 0.014 |
| SBP, mmHg | 0.047 | <0.001 |
| DBP, mmHg | -0.010 | 0.295 |
| Serum creatinine, μmol/L | -0.019 | 0.035 |
| eGFR, mL/min/1.73 m^2^ | -0.022 | 0.015 |
| HbA1c, % | 0.845 | <0.001 |
| FBG, mmol/L | 0.010 | 0.290 |
| TC, mmol/L | 0.036 | <0.001 |
| TG, mmol/L | 0.016 | 0.073 |
| LDL-C, mmol/L | 0.047 | <0.001 |
| HDL-C, mmol/L | -0.027 | 0.003 |
| hsCRP, mg/L | 0.078 | <0.001 |

BMI, body mass index; CV, cardiovascular; DBP, diastolic blood pressure; eGFR, estimated glomerular filtration rate; FBG, fasting blood glucose; HbA1c, glycosylated hemoglobin A1c; HDL-C high-density lipoprotein cholesterol; HGI, hemoglobin glycation index; hsCRP high-sensitivity C-reactive protein; LDL-C, low-density lipoprotein cholesterol; LVEF, left ventricular ejection fraction; SBP, systolic blood pressure; TC total cholesterol; TG, triglyceride.

**Table S2-**Rates of 3-Year cardiovascular outcomes stratified by HGI quintiles

| **Endpoints ^a^** | **HGI Q1**  **<-0.84**  **N = 2384** | **HGI Q2**  **[-0.84, -0.322)**  **N = 2375** | **HGI Q3**  **[-0.322, 0.075)**  **N = 2389** | **HGI Q4**  **[0.075, 0.79)**  **N = 2387** | **HGI Q5**  **≥-0.84**  **N = 2386** |
| --- | --- | --- | --- | --- | --- |
| MACE ^b^ | 79 (3.3) | 46 (1.9) | 52 (2.2) | 75 (3.1) | 75 (3.1) |
| CV death | 65 (2.7) | 32 (1.3) | 33 (1.4) | 49 (2.1) | 51 (2.1) |
| All-cause death | 90 (3.8) | 46 (1.9) | 51 (2.1) | 71 (3.0) | 68 (2.8) |
| nonfatal MI | 14 (0.6) | 14 (0.6) | 19 (0.8) | 26 (1.1) | 23 (1.0) |
| Unplanned revascularization | 140 (5.9) | 136 (5.7) | 155 (6.5) | 181 (7.6) | 187 (7.8) |

^a^ Values are presented as n (%).

^b^ MACE was defined as a composite of CV death and nonfatal MI

MACE, major adverse cardiovascular event; CV, cardiovascular; MI, myocardial infarction.

# Supplementary Figure 1- Study flowchart


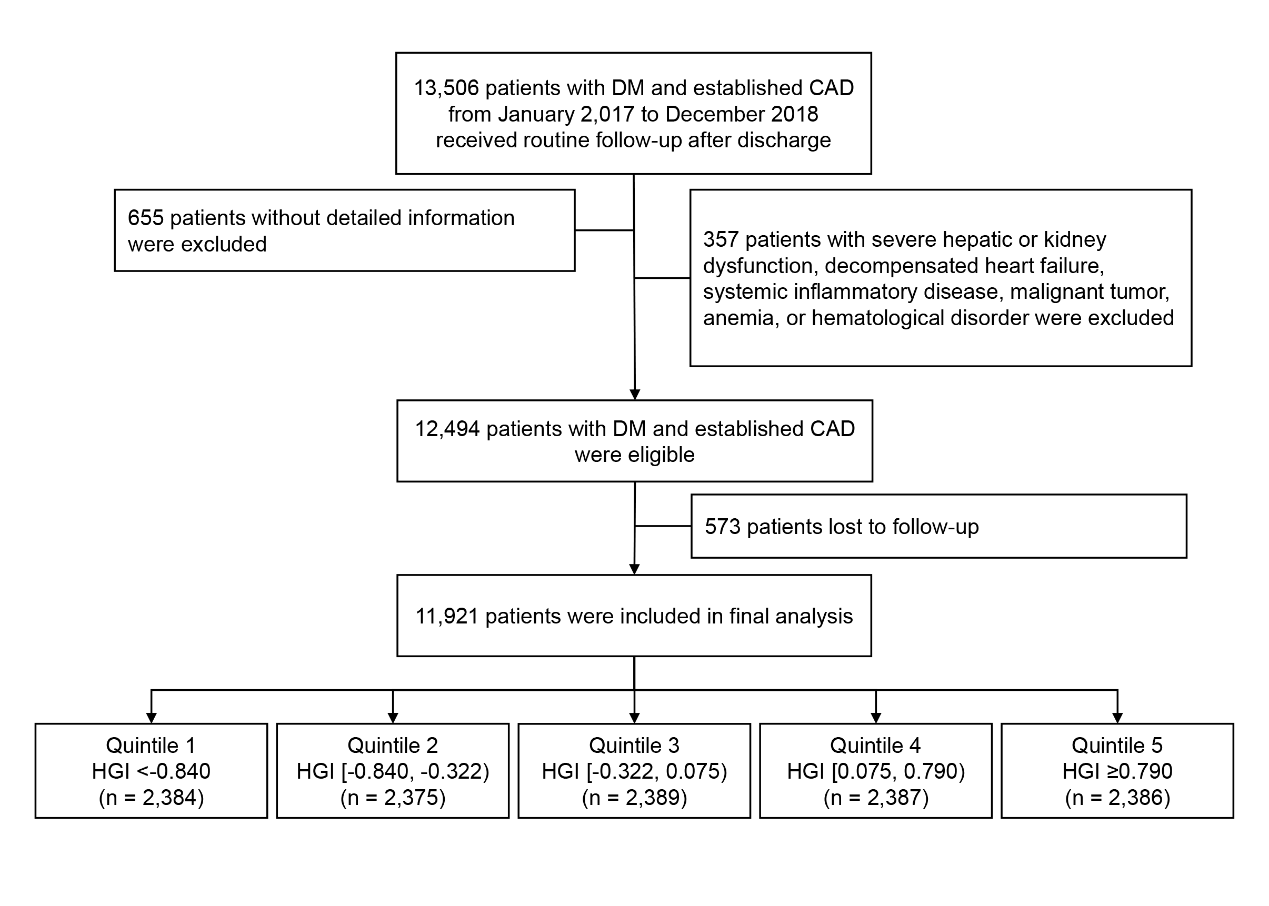


DM, diabetes mellitus; CAD, coronary artery disease

# Supplementary Figure 2-Scatter plot between HbA1c and FBG


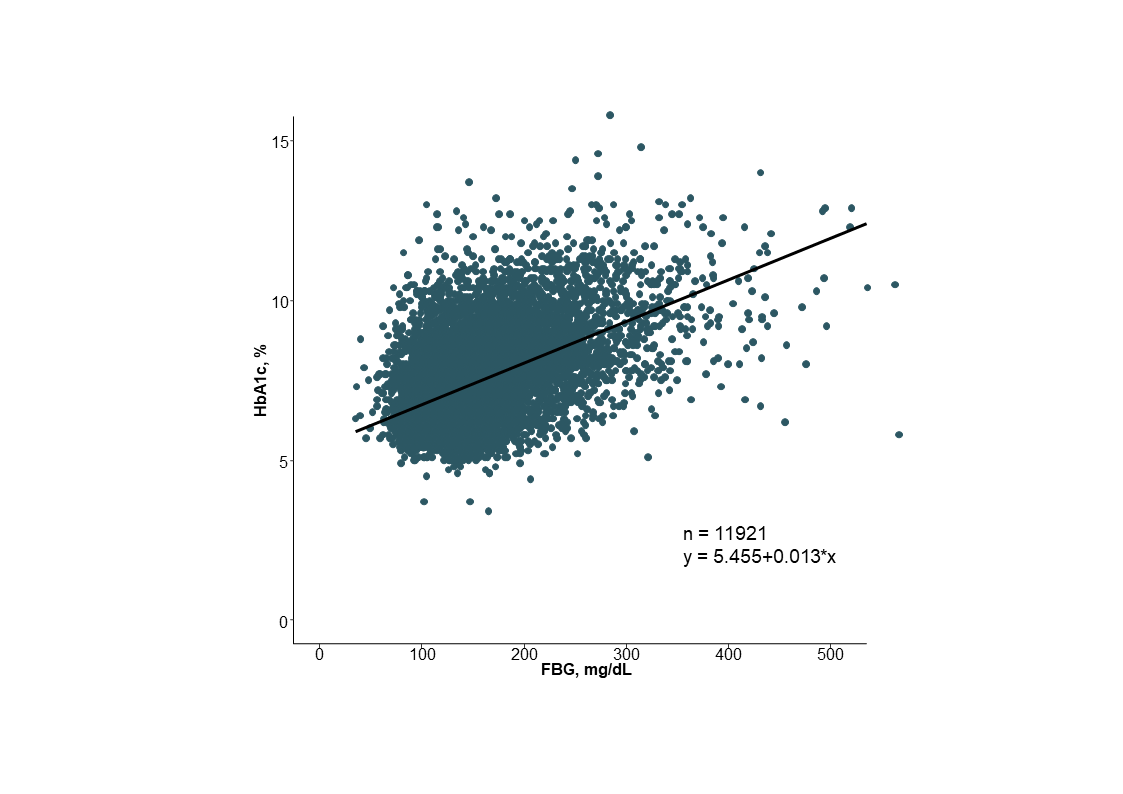


# Supplementary Figure 3-Distribution of (A) HbA1c and (B) FBG by HGI quintiles


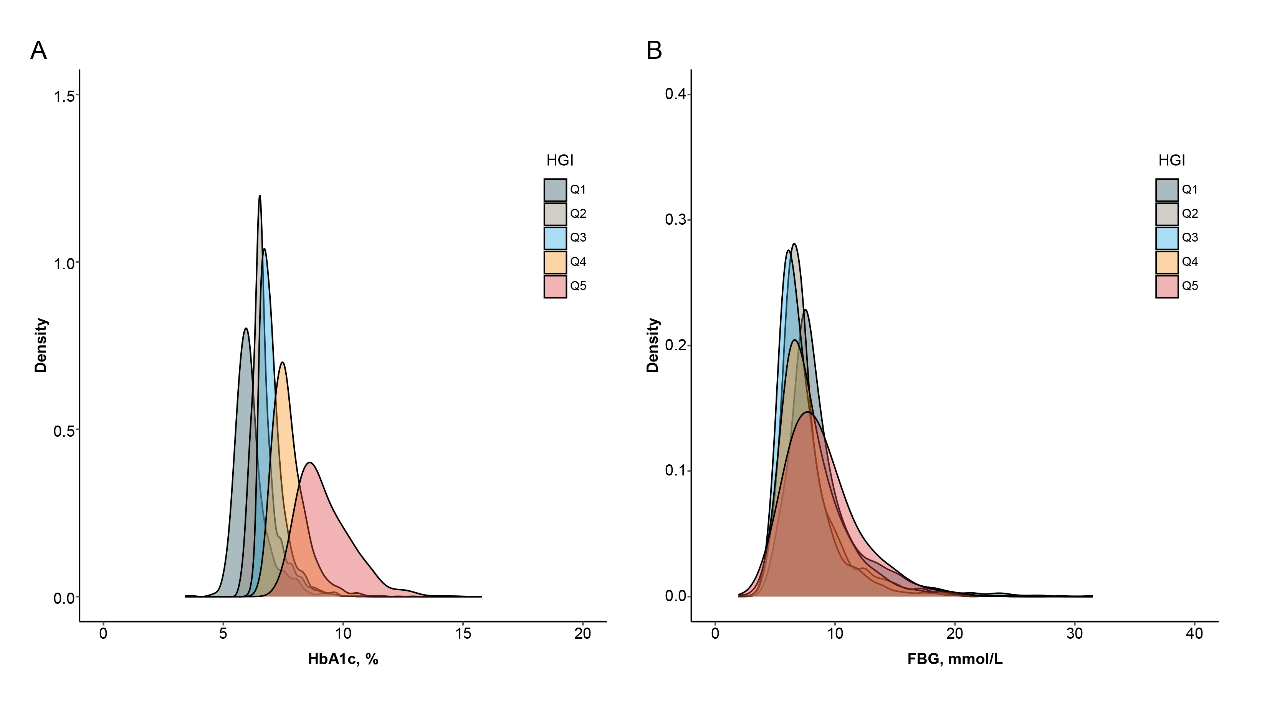

Supplement: Supplementary file 1 — Supplementary Materials [file 41387_2024_318_MOESM1_ESM.docx]
